# The Role of Cdkn1A-Interacting Zinc Finger Protein 1 (CIZ1) in DNA Replication and Pathophysiology

**DOI:** 10.3390/ijms17020212

**Published:** 2016-02-05

**Authors:** Qiang Liu, Na Niu, Youichiro Wada, Ju Liu

**Affiliations:** 1 Medical Research Center, Shandong Provincial Qianfoshan Hospital, Shandong University, 16766 Jingshi Road, Jinan 250014, China; qiangliu.sdu@gmail.com; 2 Department of Pediatrics, Shandong Provincial Hospital Affiliated to Shandong University, 324 Jingwu Road, Jinan 250021, China; judyniuna@163.com; 3 The Research Center for Advanced Science and Technology, and Isotope Science Center, The University of Tokyo, 4-6-1 Komaba, Meguro-ku, Tokyo 153-8904, Japan; wada-y@lsbm.org

**Keywords:** CIZ1, DNA replication, alternative splicing, cell cycle

## Abstract

Cdkn1A-interacting zinc finger protein 1 (CIZ1) was first identified in a yeast-2-hybrid system searching for interacting proteins of CDK2 inhibitor p21^Cip1/Waf1^. Ciz1 also binds to CDK2, cyclin A, cyclin E, CDC6, PCNA, TCF4 and estrogen receptor-α. Recent studies reveal numerous biological functions of CIZ1 in DNA replication, cell proliferation, and differentiation. In addition, splicing variants of *CIZ1* mRNA is associated with a variety of cancers and Alzheimer’s disease, and mutations of the *CIZ1* gene lead to cervical dystonia. CIZ1 expression is increased in cancers and rheumatoid arthritis. In this review, we will summarize the biological functions and molecular mechanisms of CIZ1 in these physiological and pathological processes.

## 1. Introduction

To search for p21^Cip1/Waf1^ binding proteins that regulate its subcellular localization and degradation, a yeast-2-hybrid screening, using cyclin E/p21^Cip1/Waf1^ complex as a bait, was performed and identified an unknown protein later named as CIZ1 (Cdkn1A-interacting zinc finger protein 1) [1]. CIZ1 and p21^Cip1/Waf1^ mainly stay in the nucleus when they are overexpressed separately [1]. However, when CIZ1 and p21^Cip1/Waf1^ are co-transfected into the cells, both of them translocate from the nucleus to cytoplasm [1]. In addition to p21^Cip1/Waf1^, CIZ1 has been found to interact with Cyclin-dependent kinase 2 (CDK2), Cyclin E, and Cyclin A in mouse 3T3 cell line [2], stimulating DNA replication initiation and promoting G1-S phase transition [2,3,4]. CIZ1 also exhibits DNA-binding activity [5] and acts as a co-activator of transcription factors [6]. Based on these studies, CIZ1 is proposed to function as a “mediator” that bridges cell cycle regulators during cell cycle progression. Recently, CIZ1 is reported to be associated with Alzheimer’s disease [6,7], dystonia [8], and rheumatoid arthritis [9]. In addition, elevated expression of CIZ1 was observed in many malignancies including lung cancer [10], Ewing’s tumor [11,12], colon cancer [13], gallbladder cancer [14], prostate carcinoma [15], and breast cancer [6]. In this review, we will summarize the current knowledge of the biological properties of CIZ1.

## 2. Protein Structure

**Figure 1:**
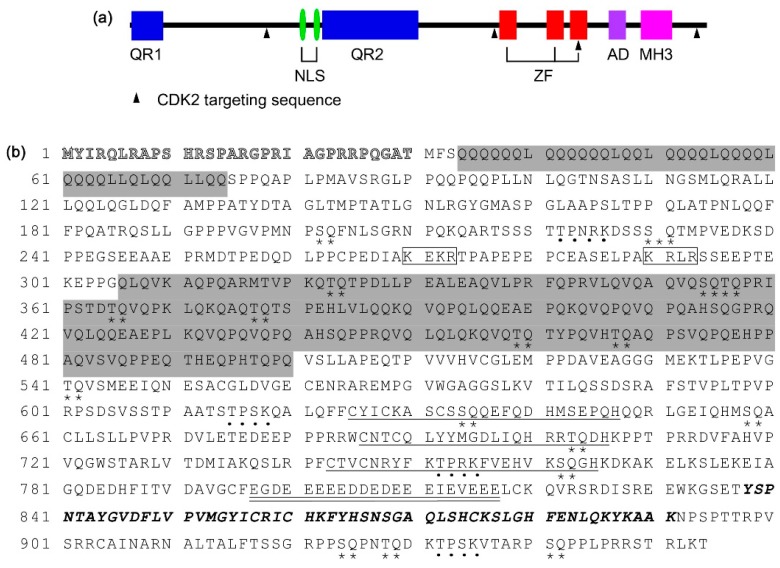
Schematic structure and sequence alignment of human CIZ1 protein. (**a**) Schematic illustration of CIZ1 protein. The glutamine rich region (QR1 and QR2), nuclear localizing signal (NLS), zinc finger domain (ZF), MH3 domain (MH3), acid domain (AD), and CDK2 targeting sequence are indicated in the diagram; and the (**b**) amino acid sequence of CIZ1 protein (Accession No. NP_001244904.1). The first 30 amino acid (aa) residues are only predicted but have not been validated [19]. The two glutamine-rich domains are shaded with grey. The three zinc finger motifs are underlined and the acidic domain is double-underlined. The MH3 domain is in bold and italic. The two nuclear localizing signals are boxed. The predicted CDK2 and PIKK phosphorylation sites are indicated by dots and asterisks, respectively.

The full-length of CIZ1 is comprised of 842 amino acid (aa) residues and contains two glutamine-rich domains, three C2H2-type zinc finger motifs, an acidic domain, and an MH3 domain [1] (Figure 1). The first glutamine-rich domain ranges from 4 to 44 aa residues and consists of 5 glutamine residue repeats separated by a single leucine residue. Unstable or abnormal expansion of glutamine residue repeats may lead to misfolding and aggregation of neurodegeneration-related proteins [16]. The second glutamine-rich domain lies between 276 to 414 aa residues, characterized by QXQ (Q: Glutamine; X: hydrophobic amino acid) triplet repeats. QXQ repeats are CIZ1-specific and have not been found in any other proteins [1]. The three zinc finger motifs, which are harbored in the C-terminus of CIZ1, range from 539 to 561, 600 to 620, and 631 to 653 aa residues [1]. A zinc finger is a basic protein structural motif, stabilized by one or more zinc icons embedded in [17]. One of the main functions of zinc finger motif is to bind DNA [18]. CIZ1 has been shown to specifically recognize and bind to a conserved DNA sequence ARYSR(0–2)YYAC (A: adenine; R: purine; Y: pyrimidine; S: either purine or pyrimidine; C: cytocine), sharing similar binding sequences with other four transcription factors, Cdx-1, GATA1, HSF, and c-Ets-1 [5]. However, whether CIZ1 is rendered with transcriptional activity is not clear.

The acidic domain of CIZ1 is composed of 21 aa residues, 18 of which are acidic aa residues [1]. Studies indicated that this domain might be related to protein interacting ability [20] and stability [21]. The MH3 domain lies between 726 and 779 aa residues of CIZ1 [1]. This domain has been found in matrin 3, a nuclear matrix protein, and NP220, a DNA-binding nuclear protein [22], suggesting that that CIZ1 may bind to DNA or nuclear matrix fractions [1]. In addition, there are two nuclear localizing signals [23] lying in N-terminus of CIZ1, and several typical CDK2 [1] and phosphatidylinositol 3-kinase-related kinase (PIKK) [24] phosphorylation sites randomly distributed in CIZ1 protein, which may have regulatory effects on CIZ1 activity and functions.

## 3. Alternative Splicing

**Figure 2:**
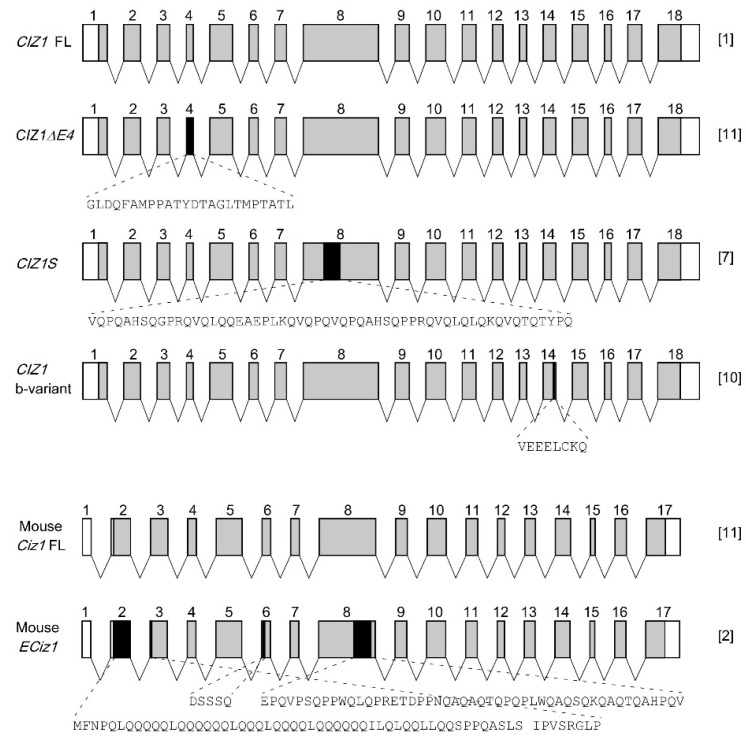
Selected alternative splicing patterns of *CIZ1* mRNA. In this diagram, we show several splicing variants, as well as the full-length of *CIZ1* mRNA of human (Accession No. NM_001257975.1) and mouse (Accession No. NM_028412.2) species. The full-length of *CIZ1* mRNA of human contains 18 exons, while that of mouse contains 17 exons. The exon numbers are indicated at the top. The alternatively spliced sequences are shaded with black. The width of the oblongs represents the relative length of the exons. The omitted amino acid tracts in the protein products due to alternative splicing are also shown in the diagram. The relative references are indicated on the right of each variant.

*CIZ1* gene localizes in 9q34 in human, comprising a DNA fragment of 38 kb. The *CIZ1* gene is composed of 18 exons and produces mRNA transcripts ranging from 2.7 to 3.2 kb due to alternative splicing. Recently, a collection of mRNA variants of *CIZ1* in human and mouse have been defined (Table 1 and Figure 2), causing diverse patterns of aa residue loss in the protein products (Figure 2). Several alternative splicing types are tissue, cell, or disease-specific. For example, variant *CIZ1ΔE4*, in which exon 4 is omitted, is found distinctly in Ewing’s tumor cells [11]. Another splicing form *CIZ1* b-variant, which lacks the last 24 nucleotides from the 3′ end of exon 14, is demonstrated to be prevalently expressed in lung tumors, but not in adjacent tissues [10]. In brain tissues of patients with Alzheimer’s disease, the variant CIZ1S level is specifically elevated in the hippocampus, and exhibits markedly higher expression level than full-length CIZ1 [7]. Thus, alternative splicing of CIZ1 enriches the biological effects of CIZ1 in various pathological processes.

**Table 1:** Alternative splicing patterns of *CIZ1* mRNA.

CIZ1 Variants	Alternative Splicing Sites	Biological Processes	References
*CIZ1ΔE4*	Exon 4	Ewing’s tumors	[11]
*CIZ1S*	Partial exon 8	Alzheimer’s disease	[7]
*CIZ1M*	Partial exon 8	Alzheimer’s disease	[7]
*CIZ1ΔE8-12*	Exon 9, 10, 11, and partial exon 8, 12	Ewing’s tumor; Primitive neuro ectodermal tumor	[12]
*CIZ1* b-variant	Exon 14	Lung cancer	[10]
Mouse *ECiz1*	Partial exon 2, 6, 8	DNA replication initiation	[2]
Mouse *Ciz1ΔE6a*	Partial exon 6	Testis development	[25]
Mouse *Ciz1ΔE4*	Exon 4	Testis development	[25]
Mouse *Ciz1ΔE4, 6a*	Exon 4 and partial exon 6	Testis development	[25]
Mouse *Ciz1ΔE3*	Exon 3	Testis development	[25]
Mouse *Ciz1ΔE3, 4*	Exon 3, 4	Testis development	[25]
Mouse *Ciz1ΔE3, 4, 6a*	Exon 3, 4 and partial exon 6	Testis development	[25]

## 4. DNA Replication

**Figure 3:**
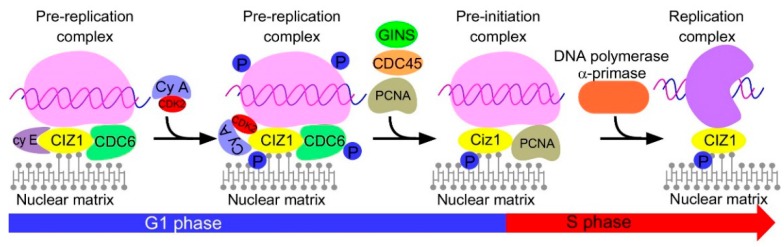
CIZ1 is involved in the initiation of DNA replication. In the early G1 phase, Ciz1 binds to Ciz1 and promotes the recruitment of CDC6, mediating the formation of the pre-replication complex. As the cell cycle progresses, cyclin A expression increases and cyclin E is replaced by cyclin A-CDK2 complex [2], which mediates phosphorylation on CIZ1, CDC6, and other components of the pre-replication complex. Phosphorylated CDC6 loses the ability to bind with CIZ1 and is substituted by PCNA [29]. Other factors, including GINS complex and CDC45, are also recruited to form a pre-initiation complex. This is followed by the binding of DNA polymerase α-primase and the DNA replication is initiated [27,28]. In this process, CIZ1 is bound to the nuclear matrix and might contribute to the stabilization of pre-replication, pre-initiation, and replication complexes [4].

The initiation of DNA replication is activated in the late G1 phase and promotes cell cycle progression from the G1 to the S phase [26]. DNA replication initiation mainly requires the involvement of two groups of initiating proteins. First, an origin recognition complex (ORC) containing ORC1-6 is assembled in the G1 phase. ORC complex can recognize and bind to the genomic DNA, determining where the DNA replication begins. Then the ORC complex recruits cell division cycle 6 (Cdc6), chromatin licensing, and DNA replication factor 1 (Cdt1), which can load a hexamer of minichromosome maintenance proteins (Mcm 2–7) to form a pre-replication complex [27,28]. Once cells undergo the late-G1 phase, a second group of proteins activate the pre-replication complex. MCM10 is recruited to the pre-replication complex and CDC6 is replaced by GINS complex and CDC45 depending on the kinase activity of CDK2 and Cdc7, triggering the activation of the pre-replication complex. Then the DNA helix is unfolded by MCM complex to recruit replication protein A (RPA) and DNA polymerase α-primase, and fire DNA synthesis [27,28]. The initiation of DNA replication is modulated by a series of regulators, e.g., cyclins, CDK2, Cdc7-dbf4, and Cdt1 inhibitor geminin [27,28]. Recent studies uncovered the many faces of CIZ1 in DNA replication initiation in mouse cell lines. First, CIZ1 is involved in the assembly of DNA pre-replication complex and replisome. CIZ1 binds to cyclin E in the G1 phase, promoting the recruitment of Cdc6 [3] and the assembly of pre-replication complex. In cells undergoing the S phase, the expression of cylin A is markedly increased and displaces cyclin E from CIZ1, which contributes to the binding of CDK2 on pre-replication complex [2]. This indicates that CIZ1 coordinates effects of cyclin E and cyclin A in the maturation of the replication complex. Cyclin A-CDK2 subsequently induces phosphorylation on 144, 192, and 293 threonine residues of CIZ1 [29] and other components of pre-replication complex [27]. Phosphorylated CIZ1 loses the capacity to interact with Cdc6 and cyclin A-CDK2, promoting the recruitment of PCNA and the activation of replisome [29]. Second, CIZ1 stabilizes the DNA pre-replication complex and replisome through the nuclear matrix binding functions. CIZ1 anchors to the nuclear matrix through the C-terminus, facilitating the stabilization of the replisome and helps to restrict the replication activity at specific sites of the chromosomes [4]. Collectively, these studies suggest CIZ1 is crucial for the initiation of DNA replication from pre-replication complex assembly to the maturation of DNA replisome (Figure 3).

## 5. Cell Proliferation and Differentiation

The assembly of pre-replication and pre-initiation complex, which is the main event in the early and late G1 phase, license G1-S phase transition in cell cycle progression [30]. As stated above, CIZ1 interacts with p21^Cip1/Waf1^, cyclin A, cyclin E, and CDK2 and promotes DNA replication initiation, indicating that CIZ1 participates in cell cycle regulation by modulating G1-S phase transition. CIZ1 also interacts with dynein light chain 1 (DLC1), a component of cytoskeleton signaling [31], to increase the activity of CDK2 and facilitate S-phase progression [32]. Inhibition of CIZ1 significantly not only reduces cell proliferation rate and the proportion of cells undergoing S phase, but also postpones the occurrence of the S phase [3]. Other studies also reveal the crucial roles of CIZ1 in the regulation of G1-S phase transition in prostate carcinoma cells [15] and RKO colorectal cancer cells [33]. In addition, the immobilization of CIZ1 in nuclear matrix is cell cycle-dependent. CIZ1 maintains at low levels during the G0/G1 phase, but significantly increases in the early S phase with a peak in the late S phase [4]. These investigations represent a critical role of CIZ1 in cell cycle progression by promoting G1-S transition.CIZ1 also participates in cell differentiation of male germ lines. It is shown that CIZ1 shows low level in mitotically-active spermatogonia and early post-mitotic spermatocytes. However, when the spermatocytes are committed to differentiate, CIZ1 is significantly up-regulated and shows high expression level in pachytene, spermatocytes, and post-meiotic spermatids [25]. Neutralization of CIZ1 using specific antibodies impairs the double-strand DNA repair capacity of testis extract [25]. These findings may suggest that the up-regulation expression of CIZ1 during spermatid generation increases the possibility of genomic DNA integrity, assuring the fidelity of genetic information.

## 6. CIZ1 and Diseases

### 6.1. Cancers

Overexpression of CIZ1 has been found in many kinds of cancer specimens and cell lines. Significantly higher expressions of CIZ1 mRNA and protein are observed in colon cancer samples than adjacent tissues, and CIZ1 level is positively correlated with a poorer survival rate [13]. In lung cancer, CIZ1 b-variant can classify 98% patients of lung cancer from normal controls [10]. An accuracy of 95% is achieved to distinguish non-small cell lung cancer from benign lung nodules by detecting CIZ1 [10]. In addition, CIZ1 expression is found to be up-regulated in gallbladder cancer [14], prostate carcinoma [15], gastric cancer [34], and undifferentiated embryonic sarcoma of the liver [35]. Consistent with CIZ1, several cell cycle regulators that can interact with CIZ1 are also involved in pathology of these cancers. Cyclin E1, one of most frequently reported cancer-related regulator, has been demonstrated to be overexpressed in lung cancer, prostate cancer, and gastrointestinal cancers [36]. P21^Cip1/Waf1^ exhibits both tumor-suppressing and oncogenic activity. Deficient expression of p21^Cip1/Waf1^ is observed in gastric cancer and non-small cell lung cancer, while overexpression of p21^Cip1/Waf1^ in prostate cancer is associated with worse clinical outcome [37]. Cyclin A1 is reported to be overexpressed in prostate cancer, gastric cancer, and lung cancer, which increases the proliferation and invasion of cancer cells [38]. CDC6, a CIZ1 partner in the formation of pre-replication complex, is down-regulated in prostate cancer [39], though CDC6 overexpression is considered to be oncogenic [40]. CDK2, which can phosphorylates CIZ1 on T174, T222 and T323 [29], is rarely found to be genetically or epigenetically altered in cancers [36]. However, the overexpresion of cyclin A and E or the down-regulation of p21^Cip1/Waf1^ may significantly increase its kinase activity in cancers [36]. Based on present studies, we speculate that CIZ1 might cooperate with cyclin E, cyclin A, p21^Cip1/Waf1^, CDC6, or CDK2 in cancer genesis or growth, and further studies are needed to confirm this viewpoint.

**Figure 4:**
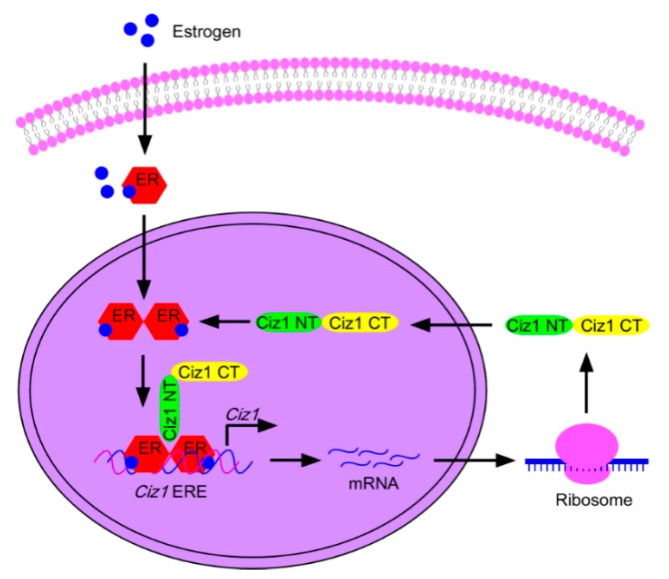
Interactions of CIZ1 and estrogen receptor signaling. Estrogen is captured by estrogen receptor-α in the cytoplasm, then the activated estrogen receptor-α is dimerized and translocates into the nucleus. CIZ1 interacts with estrogen receptor-α through the N-terminal and promotes the binding of estrogen receptor-α and the responding element, activating the transcription of targeted genes, including *CIZ1*. Estrogen-induced CIZ1 reciprocally enhances the activity of the estrogen-estrogen receptor-α signaling pathway, forming a positive feedback loop [6]. ER: estrogen receptor.

*In vitro* studies indicate that overexpression of CIZ1 leads to increased proliferation, migration, invasion, colony formation, and tumor growth in dozens of cancer cell lines [14,15,33]. Though several alternative splicing variants of *CIZ1* mRNA have been found to be associated with cancer development [10,11,12], no mutations of *Ciz1* gene have been found to be responsible for cancer genesis. Thus, CIZ1 may be more likely to be an executor rather than an oncoprotein in cancer development. Despite this, the molecular mechanisms are still not fully understood. Several studies reveal that CIZ1 is involved in signaling pathways that contribute to tumor genesis and development. Dysregulation of estrogen is one of the main risk factors for breast cancer [41]. CIZ1 binds to estrogen receptors and increases the expression of estrogen downstream target genes (Figure 4) and may contribute to the genesis of breast cancer [6]. CIZ1 also interacts with estrogen-induced protein DLC1 [42] to increase the activity of CDK2 [31], which might contribute to proliferation of breast cancer cells. In gallbladder cancer, CIZ1 is found to interact with TCF4 and positively regulate Wnt signaling [14]. Overexpression of CIZ1 increases the transcription of Wnt signaling target genes *c-Myc*, *Snail*, and *Cyclin D1*, and promotes the proliferation and migration of gallbladder cancer cells, while knockdown of CIZ1 remarkably inhibits gallbladder tumor formation in a xenograft mouse model [14]. CIZ1 is also reported to interact with p53 downstream target DNA damage-regulated gene 1 (PDRG1), a novel tumor marker that is up-regulated in cancers of colon, rectum, ovary, lung, stomach, breast, and uterus [43,44], and induces the expression of tumor-related genes *AKT* and *PSA/KLK3* [15]. Another possibility for the involvement of CIZ1 in cancer development may be due is its roles in DNA replication. Dysregulation of CIZ1 protein may mediate the disruption of DNA replication and impair the genetic fidelity of genomic DNA, which can contribute to gene mutations and cancer development. Collectively, these studies suggest that CIZ1 is an important regulator of cancer genesis and growth, and targeting CIZ1 might interrupt oncogenic signaling pathways.

Though CIZ1 is shown to promote the genesis and development of solid tumors, it exhibits distinct roles in leukemias. Exon 5 exists in all types of *CIZ1* mRNA variants and encoding a conserved CDK phosphorylation site that is essential for biological functions of CIZ1. Interestingly, *Ciz1^−/−^* mice with deletion of exon 5 develop normally, and the embryonic fibroblasts (MEFs) derived from *Ciz1^−/−^* mice do not show any defects in cell cycle progression and proliferation [45]. However, when treated with low dose of hydroxyurea, viability of *Ciz1^−/−^* MEFs is significantly impaired [45]. In addition, *Ciz1^−/−^* MEFs are more susceptible to E1A/Ras induced oncogenic transformation [45]. Moreover, MOL4070A retrovirus injection induced multiple types of leukemia in all *Ciz1^−/−^* mice tested, while none of wild-type littermates develop any type of leukemia [45] (Table 2). Since deletion of exon 5 does not affect the DNA replication functions of CIZ1, CIZ1 deficiency might induce oncogenic transformation through impairing DNA replication fidelity.

**Table 2:** CIZ1-related diseases.

Disease		Biological Behaviors of Ciz1	References
Cancers	Colon cancer	Overexpression and poorer overall survival	[13]
	Breast cancer	Estrogen responsive gene and co-activator of ER	[6]
	Lung cancer	Differentially expression of b-variant	[10]
	Prostate carcinoma	Higher expression in high stage prostate cancer	[15]
	Primitive eneuroectodermal tumor	Overexpression of variant CIZ1ΔE8-12 in cell lines	[12]
	Ewing’s tumor	Overexpression of variant CIZ1ΔE8-12 and CIZ1ΔE4	[11,12]
	Colorectal cancer	Promoting cell proliferation and colony formation	[33]
	Gallbladder cancer	Overexpression, growth and migration-promoting functions	[14]
	Gastric cancer	Overexpression	[34]
	Undifferentiated embryonal sarcoma of the liver	Overexpression	[35]
	Leukemias	anti-tumorigenesis effects	[45]
Neural diseases	Alzheimer’s Disease	Differentially expression of variant CIZ1M and involved in estrogen signaling pathway	[6,7]
	Dystonia	Point mutations	[8,46]
Autoimmune disease	Rheumatoid arthritis	Overexpression	[9]

### 6.2. Alzheimer’s Disease

In a subtractive cloning screen for novel candidate genes of Alzheimer’s disease, an alternative splicing variant of *CIZ1* mRNA in exon 8 is moderately overexpressed in the hippocampus of patients with Alzheimer’s disease, and the level of full-length *CIZ1* mRNA is not altered between the two groups [7]. Alternative splicing in exon 8 produces CIZ1 protein with the loss of 56 aa residues in the second glutamine-rich region (Table 1 and Figure 2). The shortened glutamine-rich region impairs the binding capacity of CIZ1 to the nuclear matrix, which disrupts the initiation of DNA replication during the late G1 phase and leads to G0/G1 arrest of cell cycle [7]. Dysregulated proliferation and differentiation of neural progenitor cells are one of the main causes for neuronal loss in the hippocampus of patients with Alzheimer’s disease [47]. Thus, dysfunction of CIZ1 caused by missplicing in exon 8 could participate in the pathogenesis of Alzheimer’s disease by inhibition of proliferation and differentiation of neural progenitor cells.

The risk of Alzheimer’s disease in elderly women is much higher than age-matched men, which might be the result of estrogen loss after menopause [48]. Estrogen penetrates through the membrane and binds to intracellular estrogen receptor, eliciting the expression of corresponding genes [49]. CIZ1 is identified as a novel co-activator of estrogen receptor and forms complexes with estrogen receptors through the second glutamine-rich region [6]. In addition, multiple estrogen receptor elements are identified in the *CIZ1* gene promoter and activation of estrogen signaling significantly up-regulates the expression of CIZ1 mRNA and protein level, indicating that *CIZ1* is an estrogen responsive gene [6]. These findings imply that CIZ1 might be involved in the pathogenesis of Alzheimer’s disease through a positive feedback loop of the estrogen-induced pathway.

### 6.3. Dystonia

Dystonia, which is a group of neurological movement disorders characterized by twisting and repetitive movements or abnormal postures [50], might be caused by several monogenic mutations [51]. Mutations in *CIZ1* gene are associated with dystonia (Table 2). A conserved A–G mutation in exon 7 of the *CIZ1* gene, which results in S264G substitution, is identified through exome screening of five members from a family with inherited cervical dystonia [8]. This point mutation not only alters the splicing pattern of *CIZ1* mRNA but also affects the localization of CIZ1 in nucleus [8]. CIZ1, harboring S264G substitution, forms fewer but larger particles in the nuclear fractions, which impairs the biological functions of CIZ1 [8]. Other amino acid substitution patterns, e.g., P47S, R672M, P50L, Q394E, S577F [8], and T786I [46], have also been sporadically detected. However, these mutations are extremely rare in dystonia patients. Nonetheless, another research group did not find any mutation of the *CIZ1* gene in twelve cervical dystonia families [52]. Therefore, CIZ1 may participate in the development of dystonia, but is only responsible for a small group of patients.

In addition to cancers, Alzheimer’s disease, and cervical dystonia,CIZ1 is found to be correlated with rheumatoid arthritis in a laser-mediated microdissection screening using synovial tissue samples with rheumatoid arthritis. *CIZ1* mRNA shows higher expression in rheumatoid arthritis samples than healthy controls. Further investigation shows CIZ1 protein is specifically overexpressed in the synovial sublining [9] (Table 2). Unfortunately, no further information about the roles of CIZ1 in rheumatoid arthritis has ever been gained since then.

## 7. How CIZ1 Is Regulated?

### 7.1. Alternatively Splicing

*CIZ1* mRNA can be alternatively spliced in a series of patterns as stated above (Figure 2 and Table 1), which exhibit disrupted biological behaviors compared to full-length *CIZ1*. One variant named *CIZ1ΔE4* lacks exon 4 and results in the deletion of 25 amino acid residues of the N-terminal. CIZ1ΔE4 still possesses DNA replication activity, but shows impaired ability to interact with the nuclear matrix and distributes evenly in the nucleus, while the full-length CIZ1 forms speckle-like foci with nuclear matrix [11]. As a result, CIZ1ΔE4 fails to form DNA replication foci and functions as a dominant negative inhibitor of the full-length CIZ1 in DNA replication initiation [11]. Another variant CIZ1M lacking a 28-aa residue sequence locates in the second glutamine-rich domain of N-terminus. When treated with DNase, the majority of full-length of CIZ1 is retained in the nuclear matrix fraction, while CIZ1M can hardly be detected, suggesting that the interaction between CIZ1 M and nuclear matrix is impaired [7]. Similar to CIZ1ΔE4, CIZ1M is diffused throughout the nucleus and barely forms speckled foci [7]. Although previous studies reported that the C-terminal (760–883 aa residues) is responsible for the interaction between CIZ1 and the nuclear matrix [4], these observations suggest that the subnuclear distribution of CIZ1 is directed by multiple domains outside the C-terminal.

### 7.2. Transcriptional Regulation

The *CIZ1* gene is considered to be regulated by estrogen mediated pathways and multiple sequences resembled to estrogen responsive element have been defined in the promoter of *CIZ1* gene [6]. Treatment with estrogen significantly increases the mRNA and protein level of CIZ1 in breast cancer cell lines [6]. The binding site of estrogen receptor is further confirmed in the first intron of *CIZ1* gene [6]. Interestingly, CIZ1 is also a co-activator of estrogen receptor. CIZ1 can bind to estrogen receptors with the second glutamine-rich region, enhancing the transcriptional activity of estrogen receptors [6] (Figure 4). This study suggests that CIZ1 is regulated by estrogen signaling through a positive feedback loop.

Transcription of the *CIZ1* gene is also regulated by glucocorticoid receptors. Glucocorticoid receptor is activated in the presence of cortisol or other glucocorticoids and forms homodimeric complex, which then translocates into the nucleus and binds to a specific DNA sequence named the glucocorticoid receptor element [53]. Using a chromosome conformation capture-based technique, the *CIZ1* gene is found to interact with the glucocorticoid receptor element of the *Lcn2* gene [54], a responsive target of glucocorticoid receptors [55].

As stated above, CIZ1 has been described as an estrogen receptor and glucocorticoid receptor signaling-responsive target [6,54]; thus, effectors related to these signaling pathways may be involved in the regulation of CIZ1 abundance. Estrogen and dexamethasone, which activate the estrogen receptor and glucocorticoid receptor mediated signals respectively, increase the expression of *CIZ1* mRNA [6]. However, other estrogen receptor or glucocorticoid receptor-related chemicals exhibit contrary effects. Genistein and isoflavone, structurally similar to estrogen, diminish the expression of CIZ1 mRNA and protein in a dose-dependent manner [56]. Similarly, glucocorticoid family member clobetasol inhibits *CIZ1* gene expression [57]. In addition, tyrosine kinase inhibitor AG490 significantly decreases the CIZ1 protein level [57]. These *CIZ1* gene expression inhibitors might become potential drugs for cancer therapy.

### 7.3. Phosphorylation

Though many kinases are predicted to phosphorylate on CIZ1 protein, only CDK2 has been investigated and confirmed. CDK2 mediates phosphorylation of CIZ1 at 144, 192, and 293 threonine residues [29]. Temporary phosphorylation at these sites during the late G1 phase impairs the ability of CIZ1 to form complex with cyclin E, cyclin A, and CDK2 but not CDC6, promoting the maturation of the replication complex and G1-S transition of the cell cycle [29]. However, continuous phosphorylation results in hyperphosphorylation of pre-replication complexes and failure of replisome assembly, leading to G1 arrest and proliferative inhibition [29]. Thus, phophorylation of CIZ1 regulates could regulate its functions in the initiation of DNA replication.

## 8. Conclusions and Perspectives

Since the discovery in 1999, studies of CIZ1 have achieved noticeable advances. In addition to p21^Cip1/Waf1^, CIZ1 binds with many other proteins, exerting its effects in DNA replication, cell cycle regulation, and disease development (Table 3). Dysregulation or point mutations of CIZ1 have been uncovered in neurodegenerative diseases, autoimmune diseases, and cancers, implying that CIZ1 could be considered as a diagnostic biomarker or a therapeutic target. Future studies shall focus on delineating the detailed molecular mechanisms underlying the roles of CIZ1 in pathogenesis of these diseases.

**Table 3:** CIZ1 interacting proteins.

Protein Name	Target or Binding Site on CIZ1	Biological Processes	References
p21^Cip1/Waf1^	524–670 aa residues	Cell cycle regulation	[1]
cyclin E	344RVL346	DNA replication initiation	[2]
cyclin A	344RVL346, 533KRL535	DNA replication initiation	[2]
CDK2	T174, T222, T323	DNA replication initiation	[29]
CDC6	N-terminal of CIZ1	DNA replication initiation	[29]
TCF4	Not determined	Gallbladder cancer development	[14]
Estrogen receptor-α	The 2nd glutamine-rich region	Estrogen-induced cascade	[6]
DLC1	The 2nd glutamine-rich region	G1-S transition in cell cycle	[42]
PDRG1	Not determined	Apoptosis and cell cycle regulation	[44]
